# Fucosterol from an Edible Brown Alga *Ecklonia stolonifera* Prevents Soluble Amyloid Beta-Induced Cognitive Dysfunction in Aging Rats

**DOI:** 10.3390/md16100368

**Published:** 2018-10-05

**Authors:** Jeong Hwan Oh, Jae Sue Choi, Taek-Jeong Nam

**Affiliations:** 1 Institute of Fisheries Sciences, Pukyong National University, Busan 46041, Korea; ojhwan55@pknu.ac.kr; 2 Department of Food Science and Nutrition, Pukyong National University, Busan 48513, Korea; choijs@pknu.ac.kr

**Keywords:** soluble amyloid peptide, endoplasmic reticulum stress, fucosterol, brain-derived neurotrophic factor, *Ecklonia stolonifera*

## Abstract

Fucosterol from edible brown seaweeds has various biological activities, including anti-inflammatory, anti-adipogenic, antiphotoaging, anti-acetylcholinesterase, and anti-beta-secretase 1 activities. However, little is known about its effects on soluble amyloid beta peptide (sAβ)-induced endoplasmic reticulum (ER) stress and cognitive impairment. Fucosterol was isolated from the edible brown seaweed *Ecklonia stolonifera*, and its neuroprotective effects were analyzed in primary hippocampal neurons and in aging rats. Fucosterol attenuated sAβ_1-42_-induced decrease in the viability of hippocampal neurons and downregulated sAβ_1-42_-induced increase in glucose-regulated protein 78 (GRP78) expression in hippocampal neurons via activation of tyrosine receptor kinase B-mediated ERK1/2 signaling. Fucosterol co-infusion attenuated sAβ_1-42_-induced cognitive impairment in aging rats via downregulation of GRP78 expression and upregulation of mature brain-derived neurotrophic factor expression in the dentate gyrus. Fucosterol might be beneficial for the management of cognitive dysfunction via suppression of aging-induced ER stress.

## 1. Introduction

The endoplasmic reticulum (ER), a key component of the proteostasis network, is an important organelle that plays a critical role in the correct synthesis, folding, and modification of proteins, as well as in intracellular calcium homeostasis [1,2]. Aging-induced protein damage and alteration of the redox status can cause a decrease in the folding capacity and accumulation of misfolded proteins in the ER lumen that activate a series of signaling pathways, known as the ER stress response [1,3,4,5]. Glucose-regulated protein 78 (GRP78), a member of the heat shock protein family localized in the ER lumen, is a marker of ER stress. Upon ER stress, GRP78 binds to unfolded proteins and activates a multi-chaperon complex, resulting in increased ER protein folding capacity [6]. However, severe and long-lasting ER stress, including aging-induced ER stress, can lead to the accumulation of unfolded or misfolded proteins and, subsequently, cell death. 

Age-associated decline in proteostasis is closely linked to neurodegenerative disorders, such as Alzheimer’s disease (AD) that is characterized by synaptic alteration and loss owing to the accumulation of amyloid beta (Aβ) plaques and tau neurofibrillary tangles [1]. Recent studies have suggested that soluble Aβ (sAβ) induces loss of proteostasis and triggers synaptic dysfunction, inhibition of long-term potentiation, and disruption of memory in AD [7,8,9,10,11]. These results suggest that age-induced cognitive impairment results from the dysregulation of proteostasis owing to exaggerated ER stress [12,13].

Brain-derived neurotrophic factor (BDNF) is a crucial mediator for synaptic function and plasticity that are associated with memory processing. It has been shown to play a key role in activity-induced potentiation and persistence of long-term memory in the hippocampus, the structural plasticity of the dendritic spines in rat brain, and the enhancement of neuronal protein synthesis via activation of PI3K-mToR signaling [14,15,16]. Recent studies showed that upregulation of BDNF signaling alleviated ER stress induced by cerebral ischemia or homocysteine, a risk factor for AD [17,18]. Therefore, impaired regulation of BDNF has been implicated in aging-associated psychiatric disorders [19,20,21,22]. These data indicate that activation of BDNF signaling is required for neuroprotection against aging-associated cognitive dysfunction, suggesting that BDNF-mediated regulation of ER stress could attenuate sAβ-induced neurotoxicity and aging-induced cognitive impairment.

Brown seaweeds, which are nutritional and functional food sources, are rich in fucosterol. Recently, many studies have shown that fucosterol has various biological properties, including anti-inflammatory [23], anti-adipogenic [24], antiphotoaging [25], anti-acetylcholinesterase [26], and anti-beta-secretase 1 activities [27]. Although fucosterol has a potential role in neuroprotection, little is known about its effects on sAβ-induced ER stress and cognitive impairment.

In this study, we hypothesized that fucosterol from *Ecklonia stolonifera* (*E. stolonifera*) could regulate sAβ_1-42_-induced cognitive impairment via BDNF-mediated suppression of ER stress in the dorsal hippocampus of aging rats. To verify this hypothesis, first, we determined whether fucosterol could regulate sAβ_1-42_-induced calcium dysregulation and ER stress in the primary hippocampal culture system. Next, we determined if the fucosterol-induced decrease in sAβ_1-42_-mediated ER stress was associated with the upregulation of BDNF-tyrosine receptor kinase B (TrkB)-ERK1/2 signaling. Finally using an in vivo behavioral study, we studied if fucosterol infusion attenuated sAβ_1-42_-induced memory dysfunction in the dentate gyrus of the dorsal hippocampus in aging rats.

## 2. Results

### 2.1. Fucosterol Attenuated sAβ_1-42_-Induced Decrease in the Viability of Hippocampal Neurons

**Figure 1:**
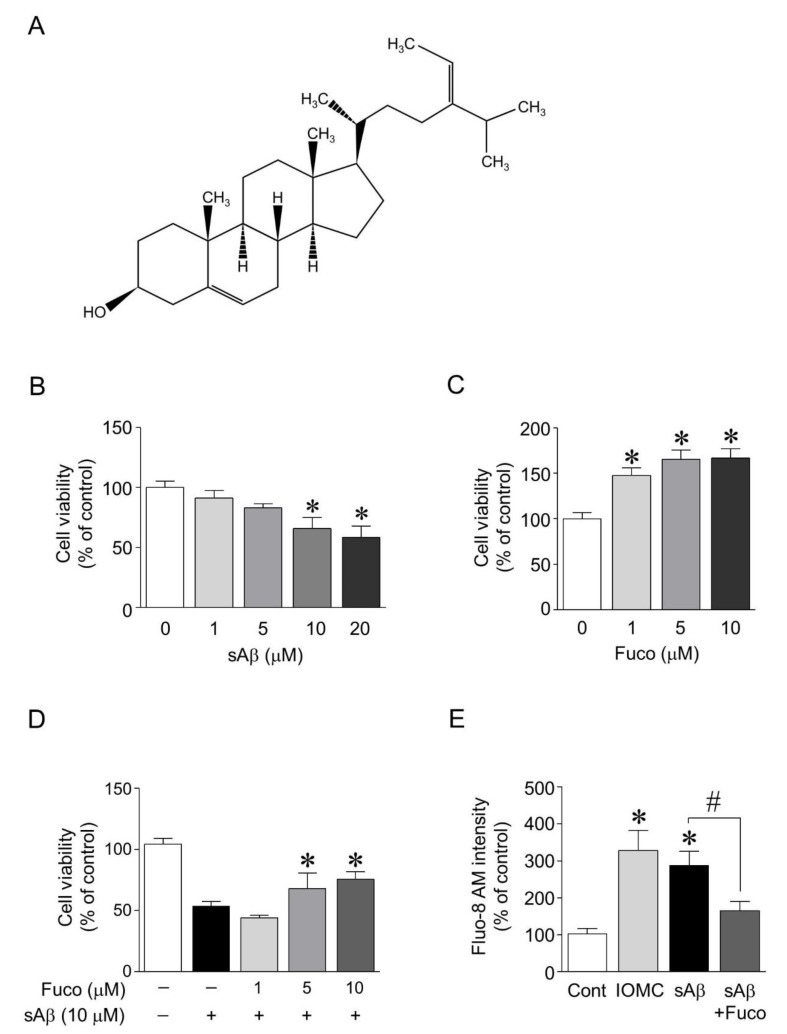
Cell viability and intracellular calcium level after sAβ_1-42_ exposure with or without fucosterol pretreatment. Fucosterol was isolated from the edible brown alga, *Ecklonia stolonifera* (**A**). Exposure to soluble amyloid beta peptide (sAβ)_1-42_ (1–20 µM) decreased the viability of rat hippocampal neurons in a dose-dependent manner. A significant difference was observed at 10 and 20 µM sAβ_1-42_ (**B**). Fucosterol (1–10 µM) significantly increased the viability of hippocampal neurons in a dose-dependent manner (**C**). Pretreatment with fucosterol (5–10 µM) significantly attenuated sAβ_1-42_-induced decrease in cell viability (**D**). sAβ_1-42_ (10 µM)-induced increase in intracellular calcium level was significantly reduced by 10 µM fucosterol pretreatment for 24 h prior to sAβ_1-42_ exposure (E). * *p* < 0.05 versus the control group; ^#^ *p* < 0.05 versus the 10 µM sAβ_1-42_ group; Cont, control; Fuco, fucosterol; sAβ, sAβ_1-42_; IOMC, ionomycin.

To investigate the protective effects of fucosterol against sAβ_1-42_-induced neurotoxicity, the doses of sAβ_1-42_ and fucosterol were first determined based on the cell viability assay. After 24 h of sAβ_1-42_ exposure (1–20 µM), the viability of hippocampal neurons (12–14 days in vitro) significantly decreased in a dose-dependent manner at 10 and 20 µM sAβ_1-42_. Fucosterol (1–10 µM) significantly increased the viability of hippocampal neurons up to 47.8% ± 14.4%, 65.5% ± 17.9%, and 66.8% ± 18.2%, respectively, as seen in Figure 1B,C. sAβ_1-42_ (10 µM)-induced decrease in cell viability was significantly attenuated by fucosterol pretreatment (5–10 µM) for 24 h prior to sAβ_1-42_ exposure, as seen in Figure 1D. Since fucosterol pretreatment attenuated sAβ_1-42_-induced decrease in hippocampal neuronal viability, we further investigated the role of fucosterol in altering intracellular calcium levels involved in ER stress. Intracellular calcium levels in hippocampal neurons were determined by the intensity of Fluo-8 AM, a cell-permeable calcium (Ca^2+^) binding dye. Ionomycin (1 µM), a Ca^2+^ ionophore raising intracellular Ca^2+^ levels, was used as a positive control because Fluo-8 calcium assay is a fluorescence-based assay for detecting intracellular calcium mobilization. As shown in Figure 1E, intracellular calcium levels significantly increased by sAβ_1-42_ (10 µM) treatment, which was downregulated by 10 µM fucosterol pretreatment for 24 h prior to sAβ_1-42_ exposure. Thus, 10 µM sAβ_1-42_ and 10 µM fucosterol were used to investigate the mechanism underlying the neuroprotective effects of fucosterol against sAβ_1-42_-induced ER stress and cognitive impairment.

### 2.2. Fucosterol Pretreatment Downregulated sAβ_1-42_-Induced Decrease in Mature BDNF Expression and Increase in GRP78 Expression in Hippocampal Neurons

**Figure 2:**
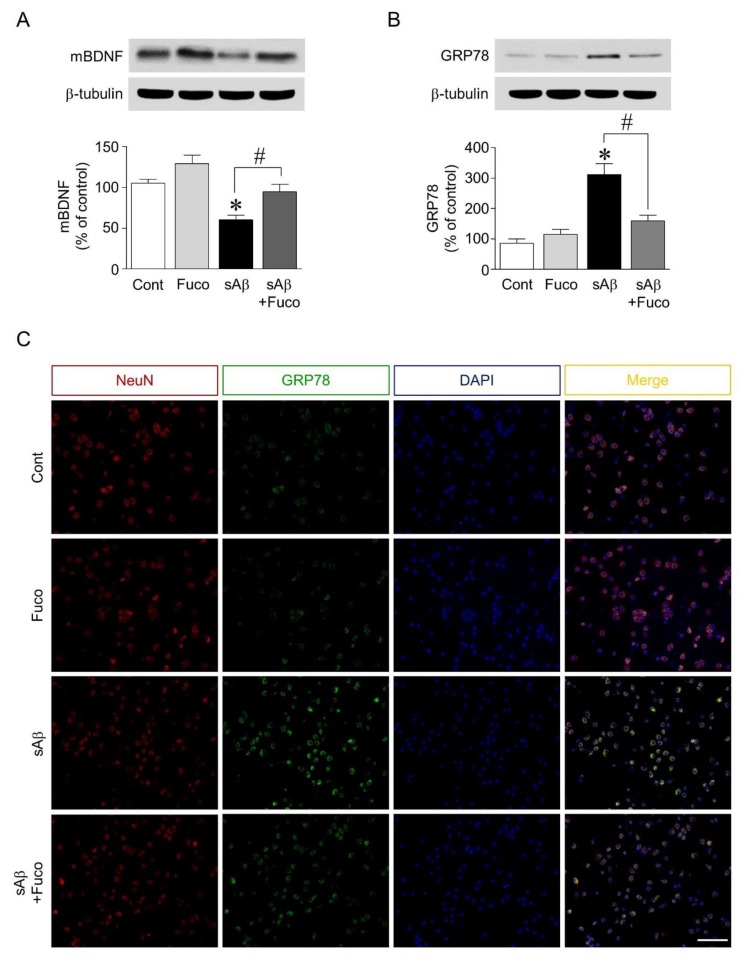
Effects of fucosterol on sAβ_1-42_-induced endoplasmic reticulum (ER) stress and decrease in brain-derived neurotrophic factor (BDNF) expression. Hippocampal neurons were treated with 10 µM sAβ_1-42_ for 24 h with or without fucosterol pretreatment (10 µM) for 24 h. sAβ_1-42_ treatment decreased the expression of mature BDNF, which was attenuated by fucosterol pretreatment (**A**). sAβ_1-42_-induced increase in the expression of glucose-regulated protein 78 (GRP78) was downregulated by fucosterol pretreatment (**B**), which was confirmed using double-immunostaining (**C**). There were no significant changes in GRP78 expression in the PYP alone group, compared to those in the control group. Scale bar represents 100 µm. * *p* < 0.05 *versus* the control group; ^#^ *p* < 0.05 *versus* the 10 µM sAβ_1-42_ group; Cont, control; Fuco, fucosterol; sAβ, sAβ_1-42_; mBDNF, mature BDNF; NeuN, neuronal nuclear antigen; DAPI, 4′,6-diamidino-2-phenylindole.

Since pretreatment with fucosterol attenuated sAβ_1-42_-induced decrease in hippocampal neuronal viability and calcium dysregulation, the potential regulation of neurotrophic factor expression and ER stress by fucosterol was further investigated. As shown in Figure 2A, sAβ_1-42_ treatment significantly decreased the expression of mature BDNF, which was attenuated by fucosterol pretreatment. ER stress was induced by exposure to sAβ_1-42_ (10 µM), which significantly decreased the viability of hippocampal neurons. ER stress was assessed by analyzing the expression levels of GRP78. sAβ_1-42_ treatment (10 µM) significantly increased GRP78 expression, which was downregulated by fucosterol pretreatment (10 µM) (Figure 2B). In the double-immunostaining analysis, as seen in Figure 2C, sAβ_1-42_-induced increase in GRP78 expression was also attenuated by fucosterol pretreatment.

### 2.3. Fucosterol Reduced sAβ_1-42_-Induced Increase in GRP78 Expression and JNK Phosphorylation Coupled to N-Methyl d-aspartate (NMDA) Receptor via Activation of TrkB-Mediated ERK1/2 Signaling

**Figure 3:**
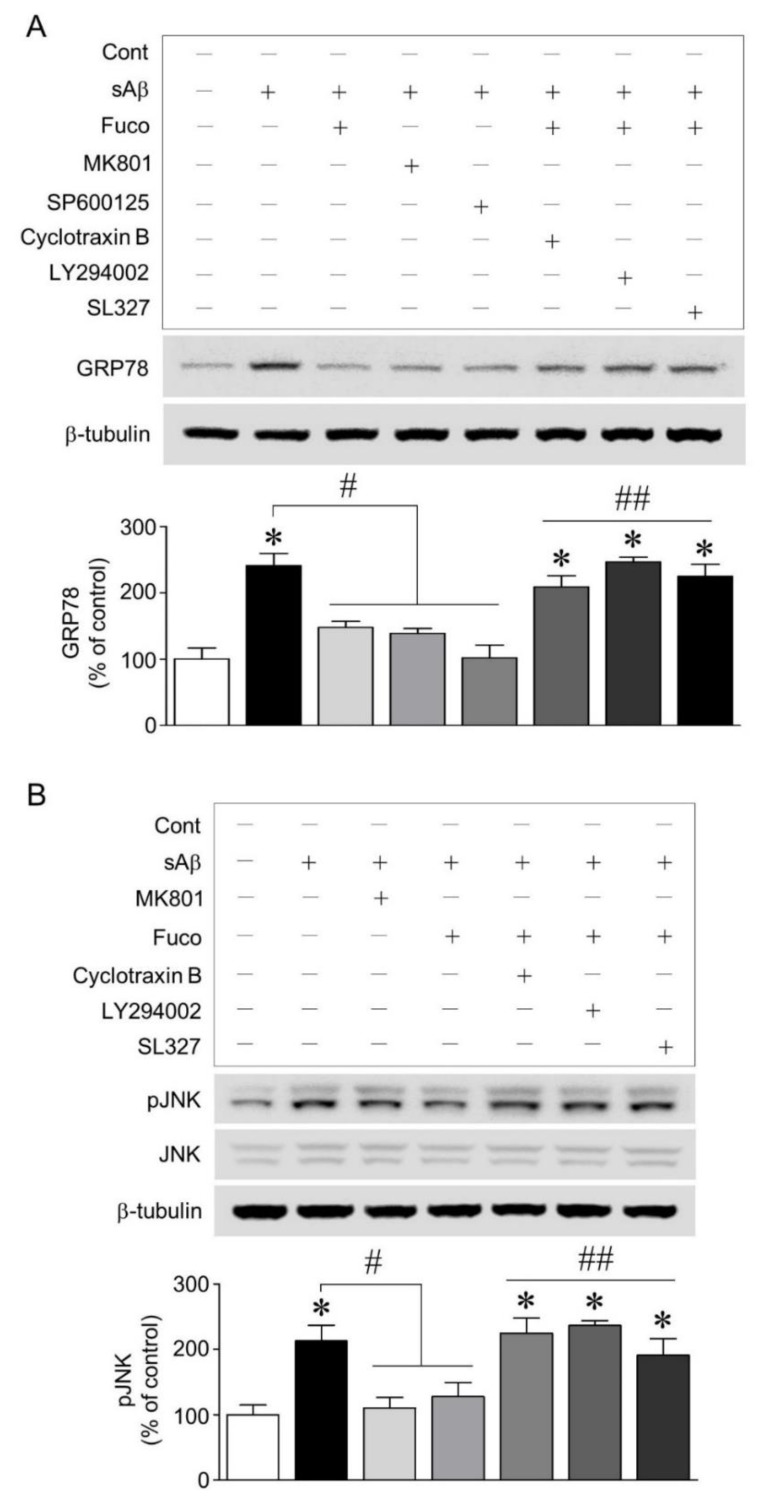
Effects of fucosterol pretreatment on sAβ_1-42_-induced GRP78 expression and the phosphorylation of JNK linked to the NMDA receptor. The blockade of NMDA receptor and the inhibition of JNK phosphorylation with MK801 (10 µM) and SP600125 (10 µM), respectively, decreased sAβ_1-42_-induced increase in GRP78 expression. Fucosterol pretreatment also significantly downregulated sAβ_1-42_-induced GRP78 expression, which was abolished by the blockade of TrkB receptor with cyclotraxin B (200 nM) and the inhibition of PI3K and ERK1/2 activation with LY294002 (20 µM) and SL327 (10 µM), respectively (**A**). sAβ_1-42_-triggered phosphorylation of JNK linked to the NMDA receptor was significantly increased in the JNK p46 isoform (46 kDa), and this increase was attenuated by fucosterol pretreatment. Fucosterol-induced decrease in JNK phosphorylation was abolished by the blockade of TrkB receptor with cyclotraxin B (200 nM) and the inhibition of PI3K and ERK1/2 activation with LY294002 (20 µM) and SL327 (10 µM), respectively (**B**). * *p* < 0.05 versus the control group; ^#^ *p* < 0.05 versus the 10 µM sAβ_1-42_ group; ^##^ *p* < 0.05 versus the 10 µM fucosterol pretreatment group; Cont, control; Fuco, fucosterol; sAβ, sAβ_1-42_.

To establish the mechanism underlying fucosterol-induced decrease in GRP78 expression, we investigated whether activation of the NMDA receptor and JNK was involved in sAβ_1-42_-induced ER stress and whether NMDA receptor-mediated ER stress was regulated by the activation of TrkB-PI3K-ERK1/2 signaling induced by fucosterol. As shown in Fig. 3A, the blockade of NMDA receptors and the inhibition of JNK phosphorylation with 10 µM MK801 and 10 µM SP600125, respectively, attenuated sAβ_1-42_ -induced increase in GRP78 expression. Furthermore, the blockade of TrkB receptors with cyclotraxin B (200 nM) and the inhibition of PI3K and ERK1/2 activation with LY294002 (20 µM) and SL327 (10 µM), respectively, 30 min prior to fucosterol pretreatment significantly abolished fucosterol-induced decrease in GRP78 expression. Since inhibition of JNK phosphorylation attenuated the increase in GRP78 expression induced by sAβ_1-42_ exposure, we also investigated whether 10 µM fucosterol pretreatment regulated JNK phosphorylation via activation of TrkB receptor-mediated ERK1/2 signaling. sAβ_1-42_ (10 µM)-induced JNK phosphorylation was significantly high in the JNK p46 isoform (46 kDa) and relatively very low in the p54 isoform (54 kDa). Additionally, these effects were attenuated by the blockade of the NMDA receptor with 10 µM MK801 and fucosterol pretreatment. Moreover, the blockade of TrkB receptor with cyclotraxin B (200 nM) and the inhibition of PI3K and ERK1/2 with LY294002 (20 µM) and SL327 (10 µM), respectively, abolished fucosterol-induced decrease in sAβ_1-42_-induced JNK phosphorylation, as seen in Figure 3B.

### 2.4. Fucosterol Co-Infusion Attenuated sAβ_1-42_-Induced Cognitive Impairment in the Dentate Gyrus of the Dorsal Hippocampus of Aging Rats

**Figure 4:**
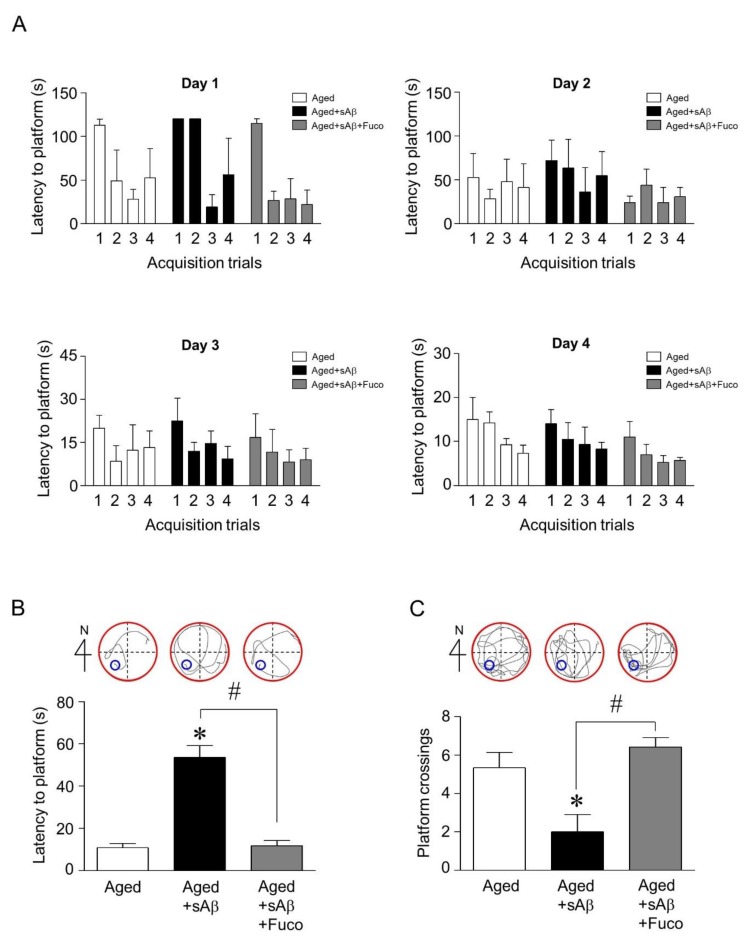
Effects of fucosterol on sAβ_1-42_-induced cognitive impairment in aging rats. sAβ_1-42_ (1 nmol) was unilaterally injected into the dentate gyrus in the dorsal hippocampus of aging rats (*n* = 6–8/group) with or without fucosterol treatment (10 µmol/h) for four weeks. The training phase was conducted for four days (**A**), and the spatial memory test was performed seven days after the last training trial. sAβ_1-42_ injection significantly increased the latency to reach the platform, which was attenuated by fucosterol co-infusion (**B**). The decrease in sAβ_1-42_-induced frequency of crossing the platform was also abolished by fucosterol treatment (C). * *p* < 0.05 *versus* control group; ^#^ *p* < 0.05 *versus* the 10 µM sAβ_1-42_; Fuco, fucosterol; sAβ, sAβ_1-42_.

Since fucosterol upregulated mature BDNF expression and attenuated sAβ_1-42_-induced decrease in GRP78 expression in hippocampal neurons, we then examined whether fucosterol could prevent sAβ_1-42_ exposure-induced cognitive dysfunction. sAβ_1-42_ (1 nmol) was unilaterally injected into the dentate gyrus of the dorsal hippocampus of aging rats (14 months) using a 0.2-µL Hamilton microsyringe. Fifteen minutes after sAβ_1-42_ injection, fucosterol was infused into the dorsal hippocampus at a rate of 10 µmol/h for four weeks using the Alzet osmotic pump. After three weeks, acquisition training was performed. On day 4 of acquisition training, latency to reach the platform decreased up to ˂30 s, as seen in Figure 4A. Thus, seven days after withdrawal, reference memory was assessed based on the latency to reach the platform and the frequency of crossing the platform. sAβ_1-42_ injection significantly increased the latency to reach the platform compared with the control, which was attenuated by fucosterol co-infusion as seen in Figure 4B. The decrease in sAβ_1-42_-induced frequency of crossing the platform was also abolished by fucosterol treatment as shown in Figure 4C.

### 2.5. Fucosterol Infusion Downregulated sAβ_1-42_-Induced Increase in GRP78 Expression and Upregulated Mature BDNF Expression in the Dorsal Hippocampus of Aging Rats

**Figure 5:**
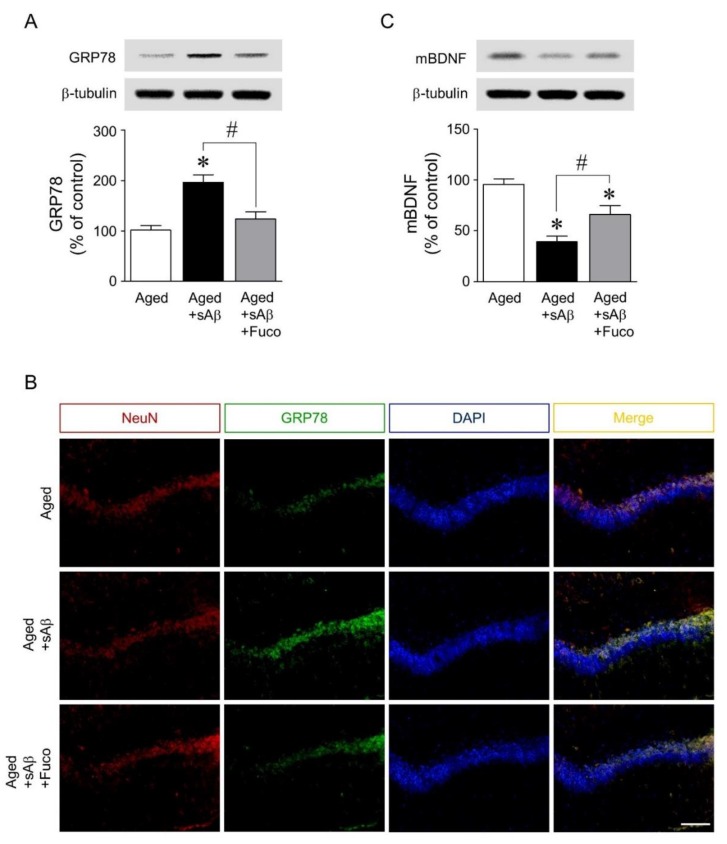
Effects of fucosterol infusion into the dentate gyrus on sAβ_1-42_-induced expression of GRP78 and mature BDNF in aging rats. sAβ_1-42_ injection significantly increased the expression of GRP78 in the dorsal hippocampus, which was attenuated by fucosterol co-infusion (**A**). Fucosterol co-infusion also downregulated sAβ_1-42_-induced increase in GRP78 immunofluorescence near the dentate gyrus of the dorsal hippocampus (**B**). In addition, sAβ_1-42_-induced decrease in mature BDNF expression was abolished by fucosterol co-infusion (**C**). Scale bar represents 100 µm. * *p* < 0.05 *versus* the control group; ^#^ *p* < 0.05 *versus* the 10 µM sAβ_1-42_ group; Fuco, fucosterol; sAβ, sAβ_1-42_; mBDNF, mature BDNF; NeuN, neuronal nuclear antigen; DAPI, 4′,6-diamidino-2-phenylindole.

Finally, the mechanisms underlying the neuroprotective effects of fucosterol against sAβ_1-42_-induced ER stress and cognitive impairment were investigated. Immediately following the Morris water maze test, rat dorsal hippocampi were dissected for Western blot analyses of mature BDNF and GRP78 expression. sAβ_1-42_ injection significantly increased the expression of GRP78 in the dorsal hippocampus, which was downregulated by fucosterol co-infusion as seen in Figure 5A. In dorsal hippocampal neurons, as shown in Figure 5B, sAβ_1-42_-induced expression of GRP78 was also attenuated by chronic fucosterol infusion. Furthermore, sAβ_1-42_-induced decrease in mature BDNF expression was abolished by fucosterol infusion, as seen in Figure 5C. 

## 3. Discussion

This study was conducted to investigate the neuroprotective effects of fucosterol against sAβ_1-42_-induced ER stress and cognitive impairment in aging rats. sAβ_1-42_ decreased hippocampal neuronal viability by increasing GRP78 expression and intracellular calcium influx; these were attenuated by fucosterol pretreatment for 24 h prior to sAβ_1-42_ treatment. In addition, sAβ_1-42_-induced ER stress was associated with the phosphorylation of JNK linked to the NMDA receptor, which was also reduced by fucosterol pretreatment via the activation of BDNF-TrkB-ERK1/2 signaling. Furthermore, a single injection of sAβ_1-42_ into the dentate gyrus of the hippocampi of aging rats increased ER stress and decreased BDNF expression and spatial memory, which were alleviated by chronic fucosterol infusion. These results showed that fucosterol decreased sAβ_1-42_-induced ER stress and cognitive dysfunction via the activation of BDNF-TrkB-ERK1/2 signaling, suggesting that ER stress-induced aging-associated cognitive dysfunction could be attenuated by fucosterol treatment.

Aging induces the dysregulation of proteostasis and the accumulation of sAβ_1-42_ that can lead to the modulation of synaptic activity and cognitive impairment [12]. Thus, a disturbance in proteostasis related to ER stress response is one of the major risk factors for neurodegenerative diseases, such as AD. Aging-induced impaired regulation of proteostasis is associated with perturbed calcium regulation that results in ER stress [28]. In this study, sAβ_1-42_ decreased the viability of hippocampal neurons and increased intracellular calcium levels, which were attenuated by fucosterol pretreatment as seen in Figure 1D,E. Calcium overload results in the dysregulation of protein phosphorylation, cytoskeletal dynamics, and gene expression, which adversely affect synaptic remodeling for memory consolidation [29]. These results indicated that fucosterol isolated from *E. stolonifera* enhanced the viability by reducing sAβ_1-42_-induced calcium overload in hippocampal neurons, suggesting that calcium-mediated ER stress could be regulated by fucosterol treatment.

As shown in Figure 2A and B, sAβ_1-42_-induced increase in the expression of GRP 78, an ER stress marker, was downregulated by fucosterol pretreatment. Fucosterol treatment also attenuated sAβ_1-42_-induced decrease in mature BDNF expression. ER stress results from the accumulation of unfolded or misfolded proteins within the ER or cytosol, and prolonged ER stress, such as aging-induced ER stress, can induce neurodegenerative diseases [28]. These results suggested that sAβ_1-42_-induced ER stress resulted from calcium dysregulation, and fucosterol pretreatment-induced decrease in ER stress was associated with the regulation of BDNF signaling.

Thus, we investigated the association between sAβ_1-42_-induced ER stress and fucosterol-induced activation of TrkB-ERK1/2 signaling that is one of the major pathways triggered by the binding of BDNF to TrkB receptors [30]. sAβ_1-42_-induced GRP78 expression was attenuated by the blockade of NMDA receptors and the inhibition of JNK phosphorylation with MK801 (10 µM) and SP600125 (10 µM), respectively, as seen in Figure 3A, suggesting that sAβ_1-42_-triggered ER stress caused was mediated by the phosphorylation of JNK linked to the NMDA receptor. sAβ_1-42_-induced increase in GRP78 expression was significantly downregulated by fucosterol pretreatment, and the effect of fucosterol was abolished by the blockade of TrkB receptors and the inhibition of PI3K and ERK1/2 activation with cyclotraxin B (200 nM), LY294002 (20 µM), and SL327 (10 µM), respectively. In addition, the fucosterol-induced decrease in the phosphorylation of JNK following sAβ_1-42_ treatment was reduced by the blockade of TrkB receptors with cyclotraxin B (200 nM) and the inhibition of PI3K and ERK1/2 activation with LY294002 (20 µM) and SL327 (10 µM), respectively, as shown in Figure 3B. Activation of TrkB signaling has been implicated in neuronal survival, synaptic plasticity, and neurodegenerative diseases [31]. These findings indicated that fucosterol could protect hippocampal neurons from sAβ_1-42_-induced ER stress through the inhibition of JNK phosphorylation via the activation of TrkB-ERK1/2 signaling, suggesting that fucosterol might prevent sAβ_1-42_-induced memory dysfunction.

**Figure 6:**
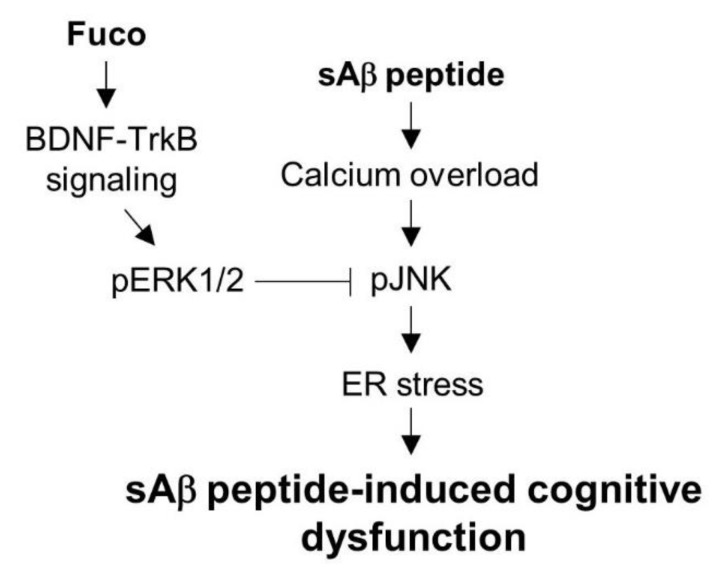
Schematic of a proposed mechanism underlying the neuroprotective effects of fucosterol from *E. stolonifera* against sAβ_1-42_-induced ER stress and cognitive impairment. sAβ_1-42_ exposure induced calcium dysregulation and ER stress, which was mediated by phosphorylation of JNK linked to NMDA receptor. Fucosterol attenuated JNK phosphorylation by sAβ_1-42_ *via* activation of TrkB-ERK1/2 signalling and the infusion of fucosterol into the dentate gyrus alleviated sAβ_1-42_-induced cognitive impairment. Fuco, fucosterol; sAβ, sAβ_1-42_.

Finally, the effects of fucosterol on sAβ_1-42_-induced memory impairment was assessed by the Morris water maze test using sAβ_1-42_-treated aging rats with or without fucosterol co-infusion. Three weeks after a single injection of sAβ_1-42_ into the dentate gyrus of aging rats, acquisition training to learn the platform position was provided for four days. Four trials per day were performed to measure the ability of rats to learn the platform place and the latencies on the 4th day were decreased up to ˂30 s, indicating that memory was formed. Following seven days of withdrawal to verify whether the memory of the target location is maintained, the probe trial was performed using a new starting position with (latency to platform) or without (crossing frequency) the submerged platform. The latency to reach the platform in sAβ_1-42_-injected aging rats without fucosterol treatment significantly increased, as seen in Figure 4B, which was attenuated by fucosterol co-infusion. The decrease in sAβ_1-42_-induced frequency of crossing platform was also abolished by fucosterol treatment as shown in Figure 4C. Furthermore, in sAβ_1-42_ injected aging rats, GRP78 expression significantly increased; however, as seen in Figure 5A,B, it was downregulated by fucosterol co-infusion. Interestingly, sAβ_1-42_ injection-induced decrease in the expression of mature BDNF was significantly recovered, but it did not reach the levels in the control group. BDNF plays a critical role in synaptic plasticity that is associated with memory processing and the persistence of long-term memory by enhancing neuronal protein synthesis [14,16]. These data suggested that sAβ_1-42_-induced cognitive impairment in aging rats could be attenuated by fucosterol via the upregulation of BDNF-TrkB-ERK1/2 signaling in the dentate gyrus as shown in Figure 6. In addition, considering the diverse functions of BDNF, the effects of fucosterol may be resulted from activation of fundamental signal cascades for neuronal viability not a specific signaling, suggesting that the fucosterol could have general neuroprotective effects against neurodegenerative diseases. Thus, our results suggested that fucosterol might have beneficial effects in controlling aging-induced ER stress and cognitive dysfunction.

## 4. Material and Methods

### 4.1. Fucosterol Extraction from E. stolonifera

Fucosterol was isolated from *E. stolonifera* collected from Tongyoung City, South Korea as previously described, and its purity (99%) was determined by HPLC [24,32]. Briefly, a lyophilized powder (500 g) of *E. stolonifera* leaf thalli was refluxed with methanol (MeOH; 3 × 3 L) for 3 h, and each filtrate was concentrated to dryness in vacuo at 40 °C to obtain the MeOH extract (116.6 g). The MeOH extract was then suspended in distilled water (H_2_O) and partitioned in sequence with dichloromethane (CH_2_Cl_2_), ethyl acetate (EtOAc), *n*-butanol (*n*-BuOH), and H_2_O to yield four fractions. The yields of the CH_2_Cl_2_ (8.27 g), EtOAc (4.17 g), *n*-BuOH (16.6 g), and H_2_O fractions (86.6 g) were 7.1, 3.6, 14.2, and 74.3%, respectively. The active CH_2_Cl_2_ (8.27 g) fraction obtained from *E. stolonifera* was separated on a silica gel chromatography column with CH_2_Cl_2_:MeOH (10:1, 10:2, and 10:3), yielding 15 subfractions (CF01-CF15). The combined fractions CF09 and CF10 were then recrystallized with CH_2_Cl_2_ and MeOH to obtain fucosterol (1390 mg). Fucosterol was identified by spectroscopic methods, including ^1^H and ^13^C NMR, as well as by comparison with published spectral data and thin-layer chromatography analysis [33]. The structure of fucosterol is shown in Figure 1. Fucosterol: white amorphous powder (CHCl_3_); m.p., 123–124 °C; EI-MS *m*/*z*: 412 [M, C_29_H_48_O]^+^, 397 [M–CH_3_]^+^, 379 [M–CH_3_–H_2_O]^+^, 314 (100) [M–C_6_H_10_O]; IR (KBr, Vmax) 3,439 (OH), 1,626 (C=C), 823 cm^-1^; ^1^H NMR (400 MHz, CDCl_3_) δ: 5.33 (1H, br d, *J* = 5.2 Hz, H-6), 5.08 (1H, q, *J* = 6.8 Hz, H-28), 3.49 (1H, m, H-3), 1.56 (3H, d, *J* = 6.8 Hz, H-29), 0.98 (3H, s, H-19), 0.95 (3H, d, *J* = 6.8 Hz, H-27), 0.95 (3H, d, *J* = 6.8 Hz, H-26), 0.92 (3H, d, *J* = 6.4 Hz, H-21), 0.66 (3H, s, H-18); ^13^C NMR (100 MHz, CDCl_3_) δ: 39.75 (C-1), 35.2 (C-2), 71.8 (C-3), 42.3 (C-4), 140.7 (C-5), 121.7 (C-6), 36.1 (C-7), 35.8 (C-8), 50.1 (C-9), 39.7 (C-10), 28.2 (C-11), 42.3 (C12), 42.3 (C-13), 56.7 (C-14), 31.6 (C-15), 34.8 (C-16), 56.7 (C-17), 11.9 (C-18), 19.4 (C-19), 39.5 (C-20), 24.3 (C21), 37.2 (C-22), 31.9 (C-23), 146 (C-24), 33.9 (C-25), 22.5 (C-26), 22.2 (C-27), 115.9 (C-28), 18.9 (C-29).

### 4.2. Primary Hippocampal Neuronal Culture

Primary hippocampal neurons were prepared from E18 rat embryos, as previously described [34]. All animal experiments were approved by the Animal Ethics Committee of Pukyong National University (approval number 2018-04) and carried out in accordance with the guidelines for the care and use of laboratory animals. Briefly, pregnant rats were deeply anesthetized with a mixture of Zoletil 50 (18.75 mg/kg) (Virbac Korea, Seoul, Korea) and Rompun (5.83 mg/kg) (Bayer Korea, Seoul, Korea), and the uteri were dissected out. The hippocampi from intact brains were dissected out in calcium-, magnesium-, and bicarbonate-free Hank’s balanced salt solution (Gibco, NY, USA) buffered with 10 mM HEPES (pH 7.3). After incubation with trypsin for 15 min at 37 °C, hippocampi were dissociated by repeatedly pipetting them up and down using a narrow tip. Then, they were plated in poly-d-lysine-coated 6-well plates at a density of 2 × 10^5^ cells/well and maintained in neurobasal medium supplemented with B27 (Gibco) at 37 °C in a humidified incubator containing 5% CO_2_. After 24 h of incubation, cytosine arabinoside at a final concentration of 5 µM was added to inhibit glial proliferation. Half of the medium was changed every three days, and the cultures were used for the experiment at 12–14 days after plating.

### 4.3. Cell Viability Assay

Cell viability was determined by Cyto X (LPS Solution, Daejeon, Korea). Cells were seeded at 2 × 10^4^ cells/well in a 96-well plate containing a final volume of 100 µL/well. They were incubated for 24 h at 37 °C in a humidified incubator containing 5% CO_2_. Soluble amyloid beta peptide (1–42, sAβ_1-42_) was prepared as previously described [35,36]. Aβ_1-42_ was purchased from Abcam (Cambridge, UK), and sAβ_1-42_ was freshly prepared in sterile water and stored at 4 °C. sAβ_1-42_ was incubated at 37 °C for 1 h before experimental use. Following exposure to sAβ_1-42_ (1–20 µM) for 24 h with or without pretreatment with fucosterol (1–10 µM) for 24 h, a water-soluble tetrazolium salt (10 µL/well) was added and incubated for 60 min at 37 °C in a 5% CO_2_ incubator. All antagonists or inhibitors were applied for 30 min before fucosterol treatment. Colored formazan was quantified by measuring the absorbance at 450 nm.

### 4.4. Intracellular Calcium Level

Hippocampal neurons were seeded at a density of 2 × 10^4^ cells/well in a 96-well plate. On day 12, the cells were incubated with 4 µM Fluo-8 AM (Abcam) for 1 h in a neuronal medium with or without fucosterol pretreatment (10 µM) for 24 h, and then exposed to 10 µM sAβ_1-42_ for 24 h at 37 °C in a 5% CO_2_ incubator. After washing twice with calcium-, magnesium-, and bicarbonate-free Hank’s balanced salt solution buffered with 10 mM HEPES (pH 7.3), changes in fluorescence were measured at excitation (480 nm) and emission wavelengths (535 nm). Ionomycin (1 µM)-treated group was used as a positive control.

### 4.5. Immunoblotting

Hippocampal neurons at a density of 2 × 10^5^ cells/well were treated with sAβ_1-42_ for 24 h with or without pretreatment with fucosterol (1–10 µM) for 24 h. The dorsal hippocampus was removed after the aging rats (14 months, 650–750 g) were deeply anesthetized with a mixture of Zoletil 50 (18.75 mg/kg; Virbac Korea) and Rompun (5.83 mg/kg; Bayer Korea). Sections were serially cut using a microtome, and the sAβ_1-42_-injected dorsal hippocampus was removed using a steel borer (inner diameter, 2 mm). Lysis of hippocampal neurons or tissue samples was performed in a radioimmunoprecipitation assay buffer containing a protease inhibitor cocktail (Thermo Fisher Scientific, MA, USA). Protein concentrations were determined using a bicinchoninic acid protein assay kit (Thermo Fisher Scientific), and proteins (20 µg) were separated using 12% sodium dodecyl sulfate-polyacrylamide gel electrophoresis. Separated proteins were transferred to a polyvinylidene difluoride membrane. The membrane was blocked with a blocking buffer containing 1% bovine serum albumin in Tris-buffered saline with 0.1% Tween 20 and then probed with primary antibodies for GRP78, JNK, phospho-JNK, and β-tubulin (Cell Signalling Technology, MA, USA) at a dilution of 1:1000 each overnight at 4 °C on a shaker. After washing three times with Tris-buffered saline with 0.1% Tween 20 for 10 min, membranes were incubated with a corresponding secondary antibody (Thermo Fisher Scientific) at a dilution of 1:10,000 for 60 min at room temperature. The membrane was stripped and reprobed with anti-β-tubulin antibody to normalize the blots.

### 4.6. Double-Immunofluorescence Staining 

Double-immunostaining was performed to confirm the expression of GRP78 in rat hippocampal neurons and dorsal hippocampus as previously described [37]. Following two washes with Dulbecco’s phosphate-buffered saline (DPBS) with Ca^2+^ and Mg^2+^, cultured neurons or brain sections (30 µm thickness) were fixed with 4% paraformaldehyde for 20 min and permeabilized with 0.3% Triton X-100 (diluted in DPBS with Ca^2+^ and Mg^2+^) for 5 min at room temperature. After three washes with PBS, the cells were incubated for 60 min at room temperature with a 5% goat serum solution in DPBS with Ca^2+^ and Mg^2+^, and then incubated overnight at 4 °C in a mixture of rabbit anti-GRP78 and mouse anti-neuronal nuclear antigen (NeuN) primary antibodies (dilution, 1:500). After washing three times with DPBS with Ca^2+^ and Mg^2+^, the cells were incubated in the mixture of two secondary antibodies (goat anti-rabbit IgG-Alexa Fluor 488 and goat anti-mouse IgG- Alexa Fluor 647) at a dilution of 1:500 for 60 min at room temperature. Following three washes and staining with 4′,6-diamidino-2-phenylindole solution for 10 min, the cells were mounted with a drop of ProLong gold anti-fade reagent (Gibco). Antibodies and normal goat serum for double-immunostaining were purchased from Abcam. The fluorescent images were taken using an EVOS^®^ FL imaging system (Thermo Fisher Scientific). 

### 4.7. Implantation of An Osmotic Pump for Sustained Fucosterol Delivery in vivo

Alzet osmotic pump (DURECT corporation, CA, USA) was used to deliver fucosterol into the dorsal hippocampus of aging rats. Rats (14 months, 650-750 g, *n* = 6–8/group) were deeply anesthetized with a mixture of Zoletil 50 (18.75 mg/kg; Virbac Korea) and Rompun (5.83 mg/kg; Bayer Korea) and placed in a stereotaxic apparatus (Stoelting Co., IL, USA). Under aseptic conditions, the Alzet brain infusion cannula attached to the flow moderator of Alzet pump model 2ML4 (DURECT corporation) was implanted into the dentate gyrus of the dorsal hippocampus using the following coordinates from the bregma (anterior-posterior, 2.28 mm; dorsal-ventral, 4.5 mm; medial-lateral, 1 mm). After covering the skull surface with dental cement and allowing it to completely harden, the Alzet pump was inserted into the subcutaneous pocket. Fucosterol was unilaterally infused into the dorsal hippocampus at a rate of 10 µmol/h for 4 weeks with or without pretreatment with sAβ_1-42_ (1 nmol) for 15 min. For the in vivo study, the dose of sAβ_1-42_ was determined based on a previous study [38].

### 4.8. Morris Water Maze Test for Spatial Learning and Memory

Following fucosterol infusion for 4 weeks, hippocampal-dependent learning and memory were assessed by the Morris water maze test. A stainless steel tank (diameter, 120 cm and depth, 45 cm) was used for the Morris water maze test. The platform was submerged 1 cm below the surface, and the water temperature was maintained at 25 °C. A set of semirandom starting positions was selected for basic acquisition training with the platform located in the southwest quadrant. Learning trials were conducted for four days (4 trials/day). Each trial was limited to 2 min, and the interval between the trials was 15 s. Seven days after the last learning trial, the reference memory was measured.

### 4.9. Statistics

Data were expressed as the mean ± standard error of the mean for each group. Statistically significant differences among groups were determined by one-way analysis of variance with repeated measures followed by Tukey’s post-hoc test using GraphPad Prism 5 (GraphPad Software, CA, USA). A *p*-value < 0.05 was considered statistically significant.

## Abbreviations

AD	Alzheimer’s disease
BDNF	Brain-derived neurotrophic factor
DPBS	Dulbecco’s phosphate-buffered saline
ER	Endoplasmic reticulum
JNK	Jun N-terminal kinase
NeuN	Neuronal nuclear antigen
NMDA	N-methyl D-aspartate
sAβ_1-42_	soluble amyloid beta peptide (1–42)
GRP78	glucose-regulated protein 78
TrkB	tyrosine receptor kinase B

## References

[1] Hetz C., Mollereau B. (2014). Disturbance of endoplasmic reticulum proteostasis in neurodegenerative diseases. Nat. Rev. Neurosci..

[2] Martínez G., Duran-Aniotz C., Cabral-Miranda F., Vivar J.P., Hetz C. (2017). Endoplasmic reticulum proteostasis impairment in aging. Aging Cell.

[3] Darling N.J., Cook S.J. (2014). The role of MAPK signalling pathways in the response to endoplasmic reticulum stress. Biochim. Biophys. Acta..

[4] Gavilán M.P., Pintado C., Gavilán E., Jiménez S., Ríos R.M., Vitorica J., Castaño A., Ruano D. (2009). Dysfunction of the unfolded protein response increases neurodegeneration in aged rat hippocampus following proteasome inhibition. Aging Cell.

[5] Zeeshan H.M., Lee G.H., Kim H.R., Chae H.J. (2016). Endoplasmic reticulum stress and associated ROS. Int. J. Mol. Sci..

[6] Gardner B.M., Pincus D., Gotthardt K., Gallagher C.M., Walter P. (2013). Endoplasmic reticulum stress sensing in the unfolded protein response. Cold Spring Harb. Perspect. Biol..

[7] Ferreira S.T., Klein W.L. (2011). The Aβ oligomer hypothesis for synapse failure and memory loss in Alzheimer’s disease. Neurobiol. Learn. Mem..

[8] Fonseca A.C., Oliveira C.R., Pereira C.F., Cardoso S.M. (2014). Loss of proteostasis induced by amyloid beta peptide in brain endothelial cells. Biochim. Biophys. Acta..

[9] Selkoe D.J., Hardy J. (2016). The amyloid hypothesis of Alzheimer’s disease at 25 years. EMBO Mol. Med..

[10] Walsh D.M., Klyubin I., Fadeeva J.V., Cullen W.K., Anwyl R., Wolfe M.S., Rowan M.J., Selkoe D.J. (2002). Naturally secreted oligomers of amyloid beta protein potently inhibit hippocampal long-term potentiation *in vivo*. Nature.

[11] Wilcox K.C., Lacor P.N., Pitt L., Klein W.L. (2011). Aβ oligomer-induced synapse degeneration in Alzheimer’s disease. Cell Mol. Neurobiol..

[12] Kaushik S., Cuervo A.M. (2015). Proteostasis and aging. Nat. Med..

[13] Scheper W., Hoozemans J.J. (2015). The unfolded protein response in neurodegenerative diseases: A neuropathological perspective. Acta. Neuropathol..

[14] Bekinschtein P., Cammarota M., Igaz L.M., Bevilaqua L.R., Izquierdo I., Medina J.H. (2007). Persistence of long-term memory storage requires a late protein synthesis and BDNF-dependent phase in the hippocampus. Neuron.

[15] Takei N., Kawamura M., Hara K., Yonezawa K., Nawa H. (2001). Brain-derived neurotrophic factor enhances neuronal translation by activating multiple initiation processes: Comparison with the effects of insulin. J. Biol. Chem..

[16] Tanaka J., Horiike Y., Matsuzaki M., Miyazaki T., Ellis-Davies G.C., Kasai H. (2008). Protein synthesis and neurotrophin-dependent structural plasticity of single dendritic spines. Science.

[17] Qiu B., Hu S., Liu L., Chen M., Wang L., Zeng X., Zhu S. (2013). CART attenuates endoplasmic reticulum stress response induced by cerebral ischemia and reperfusion through upregulating BDNF synthesis and secretion. Biochem. Biophys. Res. Commun..

[18] Wei H.J., Xu J.H., Li M.H., Tang J.P., Zou W., Zhang P., Wang L., Wang C.Y., Tang X.Q. (2014). Hydrogen sulfide inhibits homocysteine-induced endoplasmic reticulum stress and neuronal apoptosis in rat hippocampus via upregulation of the BDNF-TrkB pathway. Acta. Pharmacol. Sin..

[19] Autry A.E., Monteggia L.M. (2012). Brain-derived neurotrophic factor and neuropsychiatric disorders. Pharmacol. Rev..

[20] Boulle F., van den Hove D.L., Jakob S.B., Rutten B.P., Hamon M., van Os J., Lesch K.P., Lanfumey L., Steinbusch H.W., Kenis G. (2012). Epigenetic regulation of the BDNF gene: Implications for psychiatric disorders. Mol. Psychiatry..

[21] Erickson K.I., Miller D.L., Roecklein K.A. (2012). The aging hippocampus: Interactions between exercise, depression, and BDNF. Neuroscientist.

[22] Petzold A., Psotta L., Brigadski T., Endres T., Lessmann V. (2015). Chronic BDNF deficiency leads to an age-dependent impairment in spatial learning. Neurobiol. Learn. Mem..

[23] Jung H.A., Jin S.E., Ahn B.R., Lee C.M., Choi J.S. (2013). Anti-inflammatory activity of edible brown alga *Eisenia bicyclis* and its constituents fucosterol and phlorotannins in LPS-stimulated RAW264.7 macrophages. Food Chem. Toxicol..

[24] Jung H.A., Jung H.J., Jeong H.Y., Kwon H.J., Kim M.S., Choi J.S. (2014). Anti-adipogenic activity of the edible brown alga *Ecklonia stolonifera* and its constituent fucosterol in 3T3-L1 adipocytes. Arch. Pharm. Res..

[25] Hwang E., Park S.Y., Sun Z.W., Shin H.S., Lee D.G., Yi T.H. (2014). The protective effects of fucosterol against skin damage in UVB-irradiated human dermal fibroblasts. Mar. Biotechnol..

[26] Yoon Y.N., Chung H.Y., Kim H.R., Choi J.S. (2008). Acetyl- and butyryl-cholinesterase inhibitory activities of sterols and phlorotannins from *Ecklonia stolonifera*. Fish Sci..

[27] Jung H.A., Ali M.Y., Choi R.J., Jeong H.O., Chung H.Y., Choi J.S. (2016). Kinetics and molecular docking studies of fucosterol and fucoxanthin, BACE1 inhibitors from brown algae *Undaria pinnatifida* and *Ecklonia stolonifera*. Food Chem. Toxicol..

[28] Magi S., Castaldo P., Macrì M.L., Maiolino M., Matteucci A., Bastioli G., Gratteri S., Amoroso S., Lariccia V. (2016). Intracellular calcium dysregulation: Implications for Alzheimer’s Disease. Biomed. Res. Int..

[29] Thibault O., Hadley R., Landfield P.W. (2001). Elevated postsynaptic [Ca^2+^]_i_ and L-type calcium channel activity in aged hippocampal neurons: Relationship to impaired synaptic plasticity. J. Neurosci..

[30] Huang E.J., Reichardt L.F. (2003). Trk receptors: Roles in neuronal signal transduction. Annu. Rev. Biochem..

[31] Gupta V.K., You Y., Gupta V.B., Klistorner A., Graham S.L. (2013). TrkB receptor signalling: Implications in neurodegenerative, psychiatric and proliferative disorders. Int. J. Mol. Sci..

[32] Hwang S.W., Jang J.M., Lim S.S. (2012). Isolation of fucosterol from *Pelvetia siliquosa* by high-speed countercurrent chromatography. Fish Aquat. Sci..

[33] Lee D.G., Park J.H., Yoo K.H., Chung I.S., Lee Y.H., Lee J.K., Han D.S., Cho S.M., Baek N.I. (2011). 24-Ethylcholesta-4,24(28)-dien-3,6-dione from *Osmanthus fragrans* var. aurantiacus flowers inhibits the growth of human colon cancer cell line, HCT-116. J. Korean Soc. Appl. Biol. Chem..

[34] Kaech S., Banker G. (2006). Culturing hippocampal neurons. Nat. Protoc..

[35] Mhillaj E., Morgese M.G., Tucci P., Furiano A., Luongo L., Bove M., Maione S., Cuomo V., Schiavone S., Trabace L. (2018). Celecoxib prevents cognitive impairment and neuroinflammation in soluble amyloid β-treated rats. Neuroscience.

[36] Sotthibundhu A., Sykes A.M., Fox B., Underwood C.K., Thangnipon W., Coulson E.J. (2008). Beta-amyloid_1–42_ induces neuronal death through the p75 neurotrophin receptor. J. Neurosci..

[37] Oh J.H., Kim E.Y., Nam T.J. (2018). Phycoerythrin-derived tryptic peptide of a red alga *Pyropia yezoensis* attenuates glutamate-induced ER stress and neuronal senescence in primary rat hippocampal neurons. Mol. Nutr. Food Res..

[38] Ryu J.K., Franciosi S., Sattayaprasert P., Kim S.U., McLarnon J.G. (2004). Minocycline inhibits neuronal death and glial activation induced by beta-amyloid peptide in rat hippocampus. Glia.

